# Predictors of adherence to electronic self-monitoring in patients with bipolar disorder: a contactless study using Growth Mixture Models

**DOI:** 10.1186/s40345-023-00297-5

**Published:** 2023-05-17

**Authors:** Abigail Ortiz, Yunkyung Park, Christina Gonzalez-Torres, Martin Alda, Daniel M. Blumberger, Rachael Burnett, M. Ishrat Husain, Marcos Sanches, Benoit H. Mulsant

**Affiliations:** 1 Department of Psychiatry, Temerty Faculty of Medicine, University of Toronto, Toronto, ON Canada; 2 Campbell Family Research Institute, Centre for Addiction and Mental Health (CAMH), Toronto, ON Canada; 3 Department of Psychiatry, Dalhousie University, Halifax, NS Canada; 4 National Institute of Mental Health, Klecany, Czech Republic

**Keywords:** Bipolar disorder, Adherence, Electronic monitoring

## Abstract

**Background:**

Several studies have reported on the feasibility of electronic (e-)monitoring using computers or smartphones in patients with mental disorders, including bipolar disorder (BD). While studies on e-monitoring have examined the role of demographic factors, such as age, gender, or socioeconomic status and use of health apps, to our knowledge, no study has examined clinical characteristics that might impact adherence with e-monitoring in patients with BD. We analyzed adherence to e-monitoring in patients with BD who participated in an ongoing e-monitoring study and evaluated whether demographic and clinical factors would predict adherence.

**Methods:**

Eighty-seven participants with BD in different phases of the illness were included. Patterns of adherence for wearable use, daily and weekly self-rating scales over 15 months were analyzed to identify adherence trajectories using growth mixture models (GMM). Multinomial logistic regression models were fitted to compute the effects of predictors on GMM classes.

**Results:**

Overall adherence rates were 79.5% for the wearable; 78.5% for weekly self-ratings; and 74.6% for daily self-ratings. GMM identified three latent class subgroups: participants with (i) perfect; (ii) good; and (iii) poor adherence. On average, 34.4% of participants showed “perfect” adherence; 37.1% showed “good” adherence; and 28.2% showed poor adherence to all three measures. Women, participants with a history of suicide attempt, and those with a history of inpatient admission were more likely to belong to the group with perfect adherence.

**Conclusions:**

Participants with higher illness burden (e.g., history of admission to hospital, history of suicide attempts) have higher adherence rates to e-monitoring. They might see e-monitoring as a tool for better documenting symptom change and better managing their illness, thus motivating their engagement.

**Supplementary Information:**

The online version contains supplementary material available at 10.1186/s40345-023-00297-5.

## Background

In recent years, the wide adoption of smartphones has allowed the development of a new generation of electronic applications (apps) to monitor (e-monitor) a variety of factors directly or indirectly related to health. In the general population, several studies have examined the role of demographic factors, such as age [1–3], gender [3, 4], or socioeconomic status [1, 3, 4] and use of health apps, with age being the strongest predictor of good adherence with e-monitoring [1, 3]. In a survey, older respondents with chronic medical conditions were less likely than younger respondents to report using e-monitoring for their health [5]. However, they were more likely to report tracking some health measures (e.g., weight, blood pressure) manually and for longer periods [5]. Adherence to e-monitoring has been studied in patients with specific physical disorders: in patients with atrial fibrillation, a more severe cardiovascular disease and more atrial fibrillation episodes predicted a better adherence to e-monitoring [6]. Similarly, in patients with chronic pain, higher pain intensity and greater disability were predictors of better adherence to e-monitoring [7]; conversely, in patients with diabetes mellitus type 2, a higher Hb1Ac was associated cross-sectionally with low adherence to e-monitoring [8].

Several studies have described the feasibility and impact of e-monitoring using cell phones, computers, or smartphones, in patients with mental disorders, including bipolar disorder (BD) [9–12]. The most common goals of e-monitoring of BD patients have been to describe the longitudinal course of illness [12–15]; identify e-monitoring patterns that discriminate between different clinical states in BD [16–20]; or predict mood changes [21–24]. Despite some promising early results [25, 26], concerns have been raised about the motivation and ability of patients with BD to adhere to e-monitoring, in particular when they are depressed or manic. Some authors have expressed skepticism about the use of e-monitoring and digital technologies in individuals with severe mental illness [27–30]. For instance, following discharge from the hospital, only 44% patients with schizophrenia adhered to a mobile intervention program over 5–6 months [31]. However, to our knowledge, no study has examined clinical characteristics that might impact adherence with e-monitoring in patients with BD. Thus, we analyzed adherence to e-monitoring in patients with BD who participated in an ongoing e-monitoring study and evaluated whether demographic and clinical factors would predict adherence. Based on data in the general population or in patients with cardiovascular disease, we hypothesized that adherence would be higher in older patients and in those with more severe burden associated with BD (e.g., earlier age of onset, higher number of episodes, history of suicide attempts), and lower in those who were more symptomatic (either with depressive or manic symptoms).

## Methods

### Participants

All the participants included in the analyses enrolled in an ongoing study, the details of which have been reported previously [32]. Briefly, the study takes place in two academic hospitals in Canada: the Centre for Addiction and Mental Health, in Toronto (CAMH), Toronto, Ontario, and the Queen Elizabeth II Health Sciences Centre, Halifax, Nova Scotia. Patients referred by their treating psychiatrists were invited to consent using Informed Consent Forms approved by the research ethics boards of their hospital. All research procedures were contactless, i.e., conducted virtually.

Participants in this report were recruited between April 2021 and June 2022. In brief, they were men or women, 18 years or older (with no upper age limit), with a primary diagnosis of BD I or II according to the Diagnostic and Statistical Manual of Mental Disorders (DSM) 5 criteria [33] based on the Structured Clinical Interview for DSM-5 (SCID-5) [34], in any phase of the illness (i.e., euthymic, depressive, (hypo)manic, or mixed). Exclusion criteria include active substance use disorder according to DSM-5/SCID-5, and a mood disorder secondary to a general medical condition.

### Psychiatric Measures

Baseline assessment: after providing informed consent, participants completed a comprehensive baseline assessment that includes sociodemographic, diagnosis (SCID-5), clinical course, and pharmacotherapy. A psychiatrist administered the Young Mania Rating Scale (YMRS) [35] and the Montgomery-Asberg Depression Rating Scale (MADRS) [36] to assess severity mood symptoms. For this analysis, euthymia was operationalized as a score ≤ 10 on both scales. Specific measures are summarized in Supplementary Table 1.

Follow-up ratings: congruent with the Day Reconstruction Method [37], each day participants rated their mood, anxiety, and energy level during the previous 24 h using an electronic visual analog scale (e-VAS) accessed via a secure e-mailed link. The scale ranges from 1 (“lowest mood”) to 9 (“highest mood”), with 5 being “usual mood”. This e-VAS uses change interval of 0.1, allowing us to generate continuous, fine-grain data. Participants must complete the ratings of mood, anxiety, and energy level to submit the e-VAS. When e-VAS were missing for three days, we contacted participants by email to remind them to complete the scale daily. When data were missing for one or two days in a row, we used interpolation methods; when data were missing for three days or more, participants were not included in the e-VAS analysis.

Every week, participants completed the Patient Health Questionnaire (PHQ-9) [38] and the Altman Self-Rating Mania Scale (ASRS) [39] accessed via a secure e-mailed link. Participants must complete all items on both self-rated scales to submit their ratings. When PHQ-9 and ASRS scores were missing, we contacted participants by email to remind them the importance of filling out these scales on a weekly basis. When scores were missing for one week, we imputed them; when scores were missing for more than one week, participants were not included in the PHQ-9 and ASRS scores analysis.

Pharmacotherapy: Participants received treatment as usual from a psychiatrist. We classified current psychotropic medications into four main groups: lithium carbonate, anticonvulsants, antipsychotics, and other (e.g., antidepressants, benzodiazepines) and all models adjusted for medication groups.

### Passive Sensing (Wearable)

Participants used a wearable (ouraring.com; Oura Health Oy, Generation 2, Oulu, Finland), a waterproof titanium-made ring weighing 5 g that collects data automatically. Participants used an app to transfer their data daily to the Oura platform, where it was accessed by the research team. The ring assesses several objective variables (e.g., activity and sleep) [40] using a 3-D accelerometer and gyroscope; and physiological variables (heart rate (HR) and heart rate variability (HRV) using infrared optical pulse measurement. Oura Health Oy did not sponsor the study.

### Primary outcome measures

For each participant, we assessed adherence for: completing out the daily e-VAS (not including interpolated values); completing weekly scales (not including imputed scores); wearing the ring. To assess adherence with wearing the ring, we used the “non-wear” measure provided by the ring, which accounts for up to one hour of charging time per week. On any day, the participant was considered to be adherent with wearing the ring if the “non-wear” measure was zero, or non-adherent if the “non-wear” measure was non-zero, indicating that the participant did not wear the ring for at least part of the day. During the first eight weeks of the study, participants had access to the ring but not to the link to the daily e-VAS and weekly scale. For each participant, rates of adherence for completing e-VAS or wearing the ring were calculated as the proportion of the number of days a participant was adherent divided by the number of days between the date the participant received the invitation to fill out the e-VAS or received the mailed ring, and either June 30, 2022, or the date the participant formally withdrew from the study. A similar calculation was performed for the rate of adherence for completing the weekly scales.

### Statistical analyses

We described demographics and clinical characteristics of the sample and plotted the individual longitudinal trajectory for each of the three rates of adherence.

Growth mixture models: We identified clusters of participants based on the shapes of their adherence trajectory using a growth mixture model (GMM) implemented on the R package ‘*Flexmix*’.

Model selection: We used both the Akaike information criterion (AIC) [41] and the Bayesian Information Criterion (BIC) [42] to select the GMM best representing the observed data. For each of the three adherence rates, we tested models with 2 to 5 adherence trajectories: 2 trajectories did not allow to discriminate between adherence and non-adherence, while 5 trajectories appeared to overfit the data. Models with 3 or 4 trajectories did not differ significantly, thus we present the most parsimonious model with 3 trajectories for each of the three adherence rates. Since the AIC and BIC produced the same results, we report only the BIC in the Figures (see below). See Supplementary Table 2 for the BIC for all trajectories.

We then compared the participants’ characteristics with different adherence trajectories using ANOVA and conducted multinomial regression analyses to calculate the estimated probability of belonging to each adherence trajectory. Because we assumed that compliance changed throughout the study, we also controlled for the time in the study as a covariate in the regression analysis, using both linear and quadratic models. Lastly, we conducted cross-tabulation to compare the three rates of adherence (i.e., adherence with daily e-VAS, weekly self-rating scales, and wearing the ring). We used the Bonferroni correction test to adjust for multiple comparisons. All analyses were conducted in Mplus [43].

## Results

Eighty-seven participants were included in this analysis; Table 1 describes their clinical and demographic characteristics. Participants had an illness duration (mean ± SD) of 10.6 ± 10.1 years. Almost two-thirds of the sample (64.4%; n = 56) entered the study during euthymia; 33.3% (n = 29) entered the study during a depressive episode and 2.3% (n = 2) entered the study during a hypomanic episode. The majority of participants who entered the study euthymic remained euthymic throughout the study (82.1%; n = 46). Conversely, only 44.8% (n = 13) of those participants who entered the study during a depressive episode reached euthymia; all participants who entered the study in a hypomanic episode reached euthymia. Data collected between April 1st, 2021, and June 30th 2022 included 17,551 completed e-VAS; 2,567 weekly self-ratings; and 20,649 downloaded wearable daily data. Even though both e-VAS and wearable data are collected daily, there are fewer e-VAS datapoints because, as discussed, during the first eight weeks of the study, participants had access to the ring but not to the rating scales. Twelve (13.7%) participants withdrew from the study: two participants just after signing the informed consent; one participant after 2 weeks; eight participants after 4–6 months; and one participant after 9 months. Overall, adherence rates were 74.6% for completing the daily e-VAS, 78.5% for completing the weekly scales, and 79.5% for wearing the ring. Participants received a mean (SD) of 0.79 ± 1.63 reminders per month (median: 0; range: 0–8): 85% received 0–1 reminders; 12% received 2–5 reminders; and 3% received 6 or more reminders. Location (i.e., Toronto or Halifax) was not a significant predictor of adherence. Time in the study was not a significant covariate in the regression analyses using linear or quadratic models.

**Table 1:** Clinical and demographic characteristics of the 87 participants

Variable	
	mean ± SD
Age (years)	38.9 ± 12.4
Number of days in the study	229.4 ± 168
	**n (%)**
Sex assigned at birth (female)	59 (67.8)
Gender (woman)	55 (63.2)
Education	
Completed high school	17 (19.5)
Completed 4-year university degree	30 (34.5)
Completed post-graduate education	40 (45.9)
Marital status	
Single	46 (52.9)
Married	31 (35.6)
Divorced	10 (11.4)
Socioeconomic status	
Work full-time	44 (50.6)
Work part-time	9 (10.3)
Unemployed	11 (12.6)
On disability or social assistance	13 (14.9)
Student	7 (8.0)
Retired	3 (3.4)
Bipolar Disorder I	53 (60.9)
Predominant polarity (lifetime)	
Depressive	62 (71.3)
Manic/hypomanic or mixed	7 (8.0)
None	18 (20.7)
Polarity upon enrollment in the study	
Depressive	29 (33.3)
Manic/hypomanic	2 (2.3)
Euthymic	56 (64.4)
Rapid cycling	14 (16.1)
History of psychotic symptoms	38 (43.7)
History of suicide attempts	21 (24.1)
History of admissions	45 (51.7)
Comorbid psychiatric diagnosis(es)	67 (77.0)
Comorbid physical diagnosis(es)	29 (33.3)
Family history	
MDD	25 (28.7)
BD	23 (26.4)
Psychotic disorders	8 (9.1)
Anxiety disorders	6 (6.9)
None	3 (3.4)
Pharmacotherapy	
Lithium monotherapy	5 (5.7)
Antipsychotic monotherapy	1 (1.1)
Anticonvulsant monotherapy	1 (1.1)
Combination treatment	78 (89.7)
None	2 (2.3)

### Adherence trajectories

**Fig. 1:**
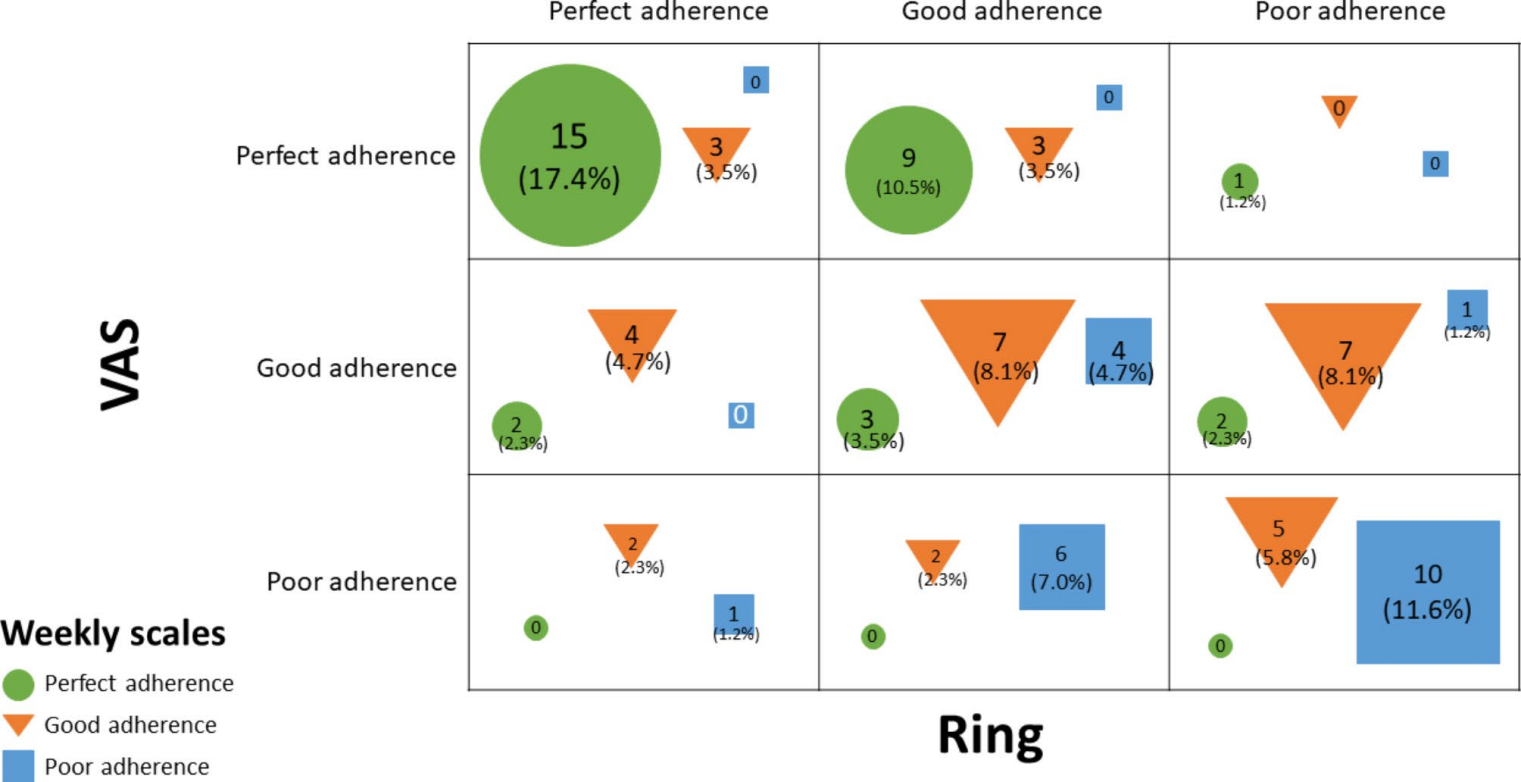
Cross-tabulation for class membership

Based on their trajectory, participants were assigned to three different groups for each metric. Inspection of the three groups revealed the following: (i) a first group of participants adherent the whole time (“perfect adherence”); (ii) a second group of participants whose adherence was good initially but declined over time (“good adherence”); and (iii) a third group of participants whose adherence rate was poor from the beginning (“poor adherence”). Figure 1 shows the cross-tabulation among the three rates of adherence (i.e., adherence with daily e-VAS, weekly self-rating scales, and wearing the rings).

One of our findings is that adherence varies with the method of data collection: some participants adhere to one or two methods but not with the other one(s). As a result, while there are only 18 (20.6%) participants with perfect adherence with all three methods, there are even fewer with poor adherence with all three methods (15 (17.2%)). Figure 1 in the paper illustrates this point but it is quite complex. Thus, we have also created a new table to show the adherence with each method (Table 2), which indicates that perfect or good adherence was comparable between the three methods (p > 0.05).

**Table 2:** Rates of adherence with each method

	Perfect	Good	Poor
Ring	27 (31.0%)	34 (39.1%)	26 (29.8%)
e-VAS	31 (35.6%)	30 (34.4%)	26 (29.8%)
Weekly scales	32 (36.7%)	33 (37.9%)	22 (25.2%)

*X*^*2*^ (2, 87) = 1.16 *p* > 0.05

### Predictors of adherence to Daily e-VAS

**Fig. 2:**
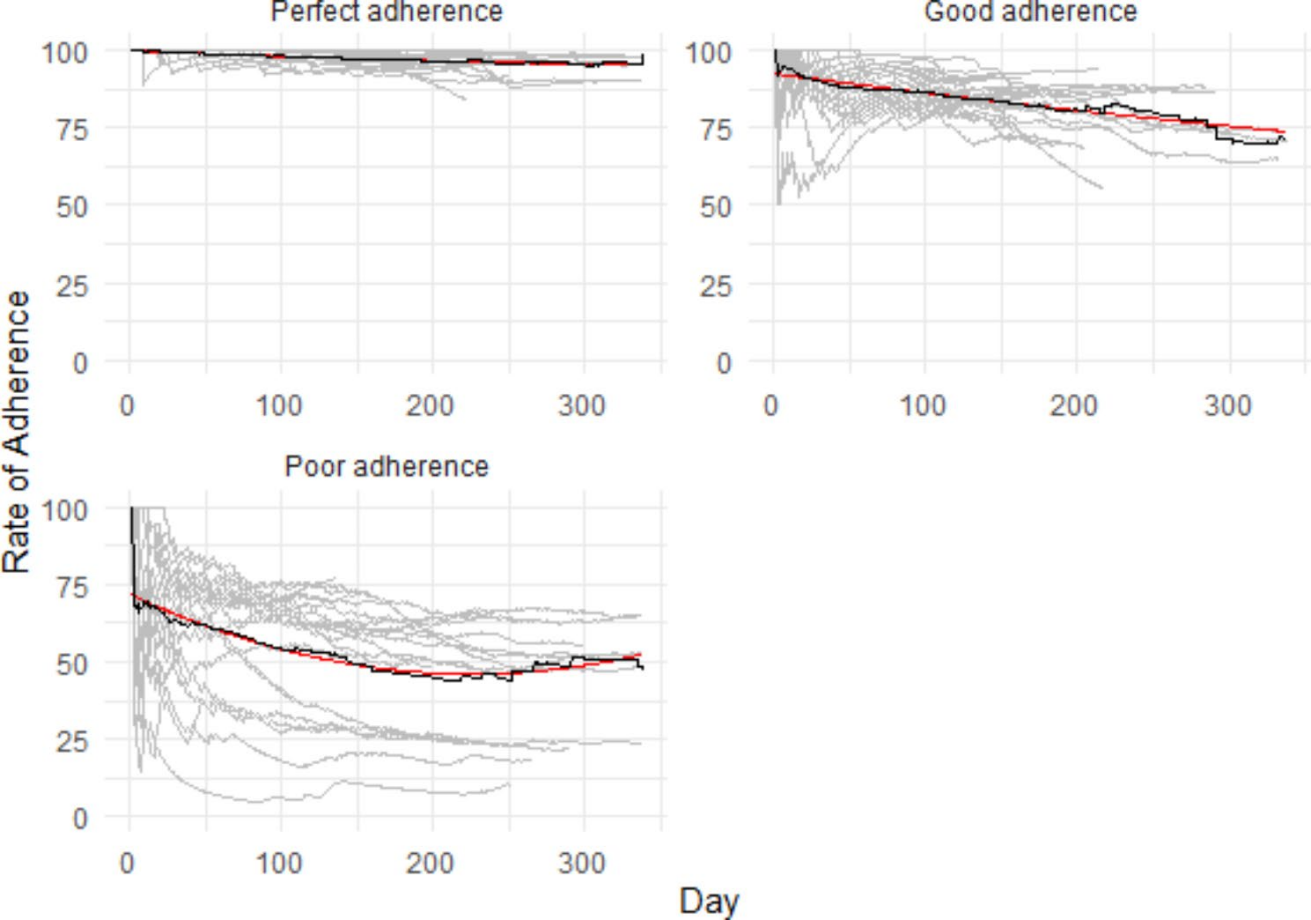
GMM for e-VAS

Figure 2 presents the adherence trajectories for e-VAS. The following characteristics were associated with a higher likelihood to be in the perfect adherence group for the e-VAS: sex (26/59 women [44.1%] vs. 5/28 [17.9%]; X^2^ = 5.68, df = 1, p = 0.01); co-morbid psychiatry diagnosis (present: 29/67 [43.3%] vs. absent: 2/20 [10.0%]; X^2^ = 7.43, df = 1, p = 0.006); and history of psychiatric admissions (present: 21/45 [46.7%] vs. absent: 10/42 [23.8%]; X^2^ = 4.94, df = 1, p = 0.02). See Supplementary Table 3 for additional information.

### Predictors of adherence to Weekly Self-Rating Scales

**Fig. 3:**
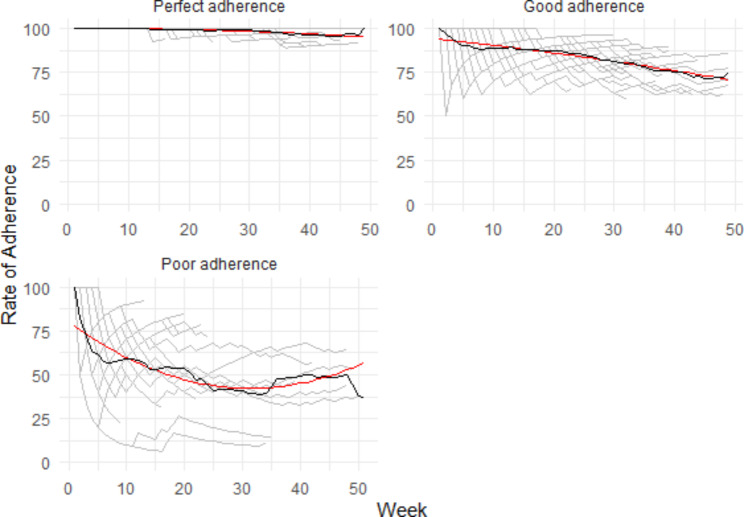
GMM for weekly self-rating scales

Figure 3 presents the adherence trajectories for weekly self-rating scales. Only sex was associated with a higher likelihood to be in the perfect adherence group for the weekly scale: sex (29/59 women [49.2%] vs. 3/28 [10.7%]; X^2^ = 12.06, df = 1, p = 0.0005). See Supplementary Table 3 for additional information.

### Predictors of adherence to Wearable

**Fig. 4:**
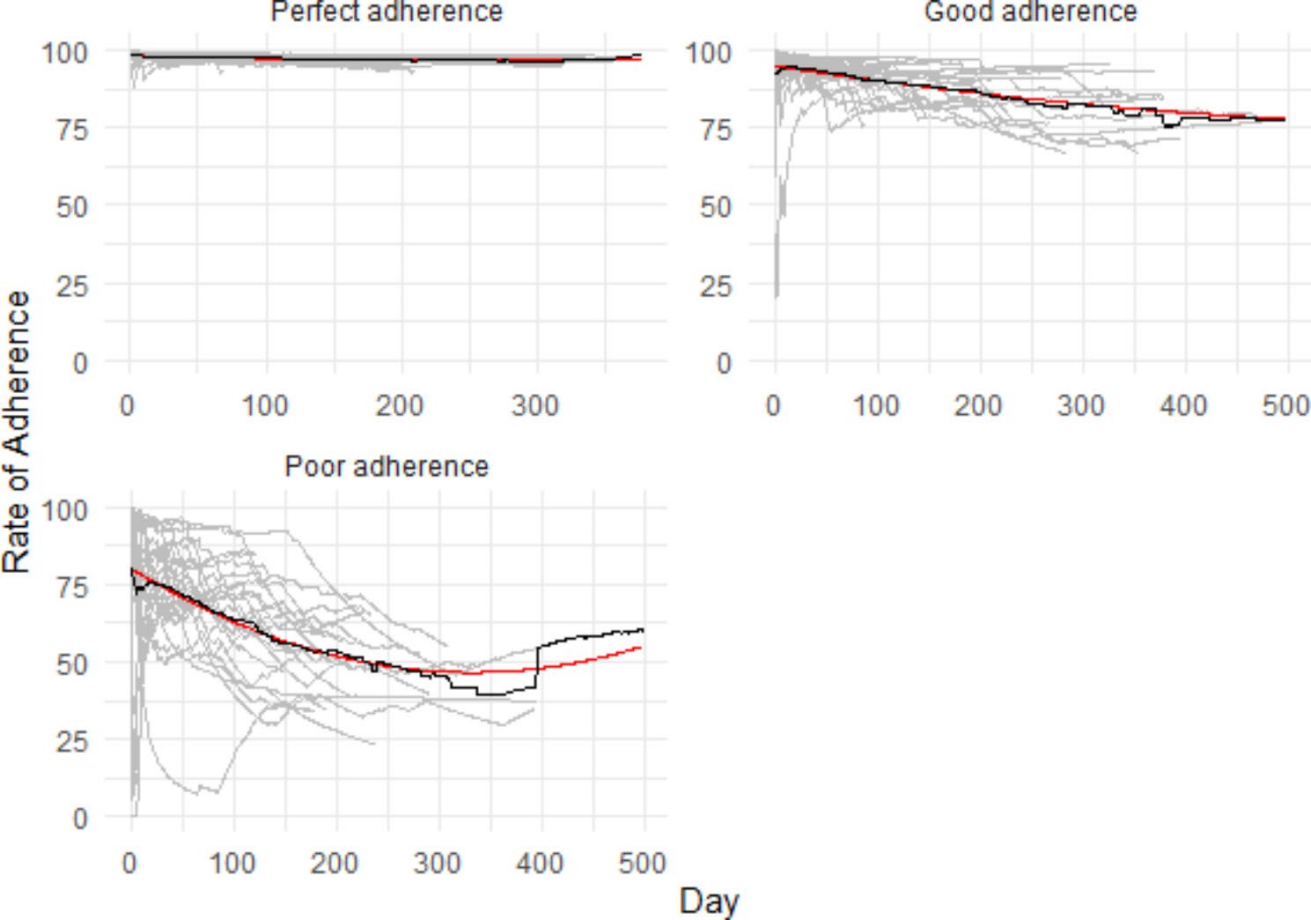
GMM for wearable

Figure 4 presents the adherence trajectories wearable data. Two characteristics were associated with a higher likelihood to be in the perfect adherence group for the wearable: sex (23/59 women [39.0%] vs. 4/28 [14.3%]; X^2^ = 5.41; df = 1, p = 0.02); and a history of suicide attempt (present: 11/21 [52.4%] vs. absent: 16/66 [24.2%]; X^2^ = 5.8935, df = 1, p = 0.01). See Supplementary Table 3 for additional information.

## Discussion

We assessed demographic and clinical predictors of adherence with self-monitoring in patients with BD who are participating in an ongoing e-monitoring study. Methodologically, our study contributes to the field by developing a robust approach for constructing adherence trajectories, which represents an important step towards understanding how participants engage with e-monitoring. Clinically, our study contributes to the literature by showing that participants with higher illness burden (e.g., history of admission to hospital, history of suicide attempts) have higher adherence rates to e-monitoring. This is important because our findings challenge current perceptions around illness burden as an obstacle to adhere to e-monitoring studies. Participants might have seen e-monitoring as a tool for better documenting symptom change and better managing their illness, thus motivating their engagement. Thus, our results contribute and expand the growing literature in e-monitoring by showing that BD patients engage with this type of studies. In this context, and although analyzing missing data was not the aim of the study, our preliminary results suggest that participants who entered the study euthymic became progressively less adherent when subsyndromal symptoms appeared, reaching their lowest adherence level upon relapse (personal communication, Halabi et al.).

Our results are also consistent with the literature showing that women are more likely to adhere to e-monitoring [3, 4] and those showing lower dropout rates in studies offering mood monitoring [44]. Moreover, our results are also consistent with those reported in participants with other physical illness, showing higher rates of adherence to e-monitoring in those patients with a more severe cardiovascular disease [6]; or with higher pain intensity [7].

One of the strengths of our study is we analyzed adherence rates to different e-monitoring metrics (wearables and self-rating scales) for over a year, using a robust methodology to cluster participants according to their adherence rates. Studies in chronic illness management emphasize the importance of measuring behavior as a component of patient adherence, as opposed to only focusing on participants’ knowledge to self-manage their illness, to improve clinical outcomes [45, 46]. Thus, in BD, along with psychoeducation to increase patients’ and families’ knowledge about the illness, we need to support the design and implementation of strategies that fill the gap between “knowing what to do” (e.g., monitor mood and sleep) and actually doing it. E-monitoring can serve as a platform to fill this gap; and thus, help improving self-management in chronic diseases, especially those that facilitate patient’s input and provider’s response in real-time [47, 48].

Finally, as suggested by many published studies, self-ratings are a useful tool for the accurate assessment and documentation of the long-term course in BD; and it is pivotal to optimizing treatment and improving the outcome of each individual patient [49, 50]. Moreover, in the context of analyzing mood variability, self-ratings are not only clinically relevant, but they provide a richer framework on which to understand mood variability and treatment response [51, 52].

Limitations include: a small sample size; while polarity upon entrance to the study was not associated with adherence to any of the three metrics, there are only two participants who entered the study hypomanic, for which is difficult to assess the effect of this particular polarity on adherence. While it has been proposed that insight fluctuates throughout the course of illness in BD [53], and this could potentially affect adherence rates, we did not include this measure in our study. Finally, while we did not find that time in the study was a significant variable, a more fine-grained picture of the correlation between clinical improvement or deterioration and missing data throughout the study is missing. Although some authors have reported that almost one quarter of patients diagnosed with schizophrenia participating in remote monitoring were not adherent to the study [54], it is unclear whether this was in the context of an acute illness exacerbation. This is an active area of study that deserves further attention.

Future studies should include the development and deployment of interventions to improve adherence in those with decreasing or partial adherence, in order to design better, personalized tools to improve self-management of the illness [55]. In this context, Just-In-the-Moment Adaptive Interventions (JITAIs), might be useful. A JITAI, an intervention design that adapts the provision of support, with the goal to deliver support “at the moment and in the context that the person needs it most and is most likely to be receptive” [56] are driven by the importance of capitalizing on periods of heightened susceptibility to positive behavior changes [57]. While, to our knowledge, there are no comprehensive studies on JITAI in bipolar disorder, we believe that future studies could capitalize on our findings by intervening when participants enter the study in a depressive episode and thus, design tailored strategies (e.g., psychoeducation) when they are the most receptive.

## Conclusions

Our study adds knowledge to the field by developing a robust methodology to cluster participants according to their adherence rates; and by showing that those patients who are clinically at the highest risk of relapse showed the highest adherence to e-monitoring. Furthermore, the comprehensive approach used in this study to identify clinical characteristics associated with adherence trajectories will allow for a more sophisticated approach to the use of e-monitoring in mood disorders.

## Electronic supplementary material

Below is the link to the electronic supplementary material.

Supplementary Material 1

Supplementary Material 2

Supplementary Material 3

## Data Availability

The data that support the findings of this study are available on request from the corresponding author. The data are not publicly available due to privacy or ethical restrictions.
