# Supplementary material for: Predictors of adherence to electronic self-monitoring in patients with bipolar disorder: a contactless study using Growth Mixture Models
Source: Int J Bipolar Disord. 2023 May 17;11:18. doi: 10.1186/s40345-023-00297-5 (PMC10192477; doi:10.1186/s40345-023-00297-5)
Supplement: Supplementary file 1 — Supplementary Material 1 [file 40345_2023_297_MOESM1_ESM.docx]

| **Domain** | **Instrument** | **Main variables** |
| --- | --- | --- |
| Socio-Demographics | Socio-demographic questionnaire | Self-reported gender, race/ethnicity, age, occupation, number of years of education, marital and work status |
| Diagnosis | SCID-5, MADRS, YMRS | Diagnosis of BD I or II; polarity upon entrance to the study |
| Clinical Course | Clinical questionnaire | Age at onset, number and type of previous episodes, history of suicide attempts, history of psychotic symptoms during episodes, co-morbid disorders, number of lifetime admissions, family history of any psychiatric disorder in first-and second-degree relatives |
| Pharmacotherapy | Clinical questionnaire | Name, dosage, and date medication(s) started |
| Daily self-ratings | VAS | Mood, energy, and anxiety levels |
| Weekly self-ratings | PHQ-9 and ASRS | Self-rating measuring presence of depressive or (hypo)manic symptoms |

**Supplementary Table 1: Sociodemographic and clinical variables collected**

ASRS: Altman Self-Rating Mania Scale; BD: Bipolar disorder; MADRS: Montgomery-Asberg Depression Rating Scale; PHQ-9: Patient Health Questionnaire, 9 items;SCID: 5: Structured Clinical Interview for DSM-5; YMRS: Young Mania Rating Scale; VAS: Visual Analog Scale.
