# Supplementary material for: Predictors of adherence to electronic self-monitoring in patients with bipolar disorder: a contactless study using Growth Mixture Models
Source: Int J Bipolar Disord. 2023 May 17;11:18. doi: 10.1186/s40345-023-00297-5 (PMC10192477; doi:10.1186/s40345-023-00297-5)
Supplement: Supplementary file 2 — Supplementary Material 2 [file 40345_2023_297_MOESM2_ESM.pdf]

Table 2 Fit Indices for unconditional 2- to 5-class growth mixture models

(a) Ring

**deg \ class**	**2-class**	**3-class**	**4-class**	**5-class**
2	(-4.17,-4.16)	(-4.96,-4.96)	(-5.33,-5.32)	(-5.50,-5.49)
3	(-4.18,-4.18)	(-4.97,-4.96)	(-5.35,-5.34)	(-5.52,-5.50)
4	(-4.19,-4.18)	(-4.97,-4.96)	(-5.36,-5.35)	(-5.54,-5.53)
5	(-4.19,-4.18)	(-4.97,-4.96)	(-5.35,-5.34)	(-5.51,-5.50)

† (AIC, BIC)

(b) VAS

**deg \ class**	**2-class**	**3-class**	**4-class**	**5-class**
2	(5.60,5.60)	(4.85,4.85)	(4.39,4.39)	(3.99,4.00)
3	(5.60,5.61)	(4.84,4.85)	(4.39,4.40)	(3.96,3.98)
4	(5.60,5.61)	(4.84,4.85)	(4.39,4.40)	(3.96,3.97)
5	(5.60,5.60)	(4.84,4.85)	(4.39,4.40)	(3.99,4.01)

† (AIC, BIC)

(c) Weekly scales

**deg \ class**	**2-class**	**3-class**	**4-class**	**5-class**
2	(5.63,5.65)	(4.70,4.73)	(4.27,4.31)	(-5.47,-5.41)
3	(5.41,5.44)	(4.69,4.73)	(4.26,4.31)	(-5.49,-5.43)
4	(5.62,5.65)	(4.67,4.71)	(-5.23,-5.16)	(-5.32,-5.24)
5	(5.61,5.65)	(4.65,4.70)	(4.21,4.28)	(-5.53,-5.44)

† (AIC, BIC)
