# Supplementary material for: Predictors of adherence to electronic self-monitoring in patients with bipolar disorder: a contactless study using Growth Mixture Models
Source: Int J Bipolar Disord. 2023 May 17;11:18. doi: 10.1186/s40345-023-00297-5 (PMC10192477; doi:10.1186/s40345-023-00297-5)
Supplement: Supplementary file 3 — Supplementary Material 3 [file 40345_2023_297_MOESM3_ESM.docx]

Supplementary Table 3. e-VAS, weekly self-rating scales and wearable adherence trajectories

| **Variable** | **e-VAS: Perfect** | **e-VAS: Good** | **e-VAS: Poor** | **e-VAS p-value** | **Weekly: Perfect** | **Weekly: Good** | **Weekly: Poor** | **Weekly p-value** | **Ring: Perfect** | **Ring: Good** | **Ring: Poor** | **Ring p-value** |
| --- | --- | --- | --- | --- | --- | --- | --- | --- | --- | --- | --- | --- |
| n | 31 | 30 | 26 |  | 32 | 33 | 22 |  | 27 | 34 | 26 |  |
| Age (mean ± SD) | 39.9 ± 11.4 | 39.6 ± 13.1 | 37 ± 13.1 | 0.631 | 40.4 ±12.5 | 40.9 ± 12.7 | 33.9 ± 11.1 | 0.089 | 40.3 ± 13.8 | 38.8 ± 11.6 | 37.8 ± 12.3 | 0.767 |
| Sex | | | | | | | | | | | | |
| Male | 5 (16.1) | 11 (36.7) | 12 (46.2) | **0.001** | 3 (9.4) | 18 (54.5) | 7 (31.8) | **0.001** | 4 (14.8) | 10 (29.4) | 14 (53.8) | **0.02** |
| Female | 26 (83.9) | 19 (63.3) | 14 (53.8) |  | 29 (90.6) | 15 (45.5) | 15 (68.2) |  | 23 (85.2) | 24 (70.6) | 12 (46.2) |  |
| Education | | | | | | | | | | | | |
| <12 years | 7 (22.6) | 7 (23.3) | 3 (11.5) | 0.469 | 9 (28.1) | 5 (15.2) | 3 (13.6) | 0.302 | 7 (25.9) | 7 (20.6) | 3 (11.5) | 0.410 |
| ≥ 12 years | 24 (77.4) | 23 (76.7) | 23 (88.5) |  | 23 (71.2) | 28 (84.8) | 19 (86.4) |  | 20 (74.1) | 27 (79.4) | 23 (88.5) |  |
| Marital status | | | | | | | | | | | | |
| Married | 12 (38.7) | 11 (36.7) | 8 (30.8) | 0.815 | 13 (40.6) | 12 (36.4) | 6 (27.3) | 0.599 | 10 (37.0) | 14 (41.2) | 7 (26.9) | 0.512 |
| Single or divorced | 19 (61.3) | 19 (63.3) | 18 (69.2) |  | 19 (59.4) | 21 (63.6) | 16 (72.7) |  | 17 (63.0) | 20 (58.8) | 19 (73.1) |  |
| Socioeconomic status | | | | | | | | | | | | |
| Working | 18 (58.1) | 19 (63.3) | 16 (61.5) | 0.912 | 19 (59.4) | 22 (66.7) | 12 (54.5) | 0.649 | 15 (55.6) | 20 (58.8) | 18 (69.2) | 0.565 |
| Not working | 13 (41.9) | 11 (36.7) | 10 (38.5) |  | 13 (40.6) | 11 (33.3) | 10 (45.5) |  | 12 (44.4) | 14 (41.2) | 8 (30.8) |  |
| Primary diagnosis | | | | | | | | | | | | |
| BD Type I | 21 (67.7) | 17 (56.7) | 15 (57.7) | 0.623 | 23 (71.9) | 16 (48.5) | 14 (63.6) | 0.148 | 17 (63.0) | 25 (73.5) | 11 (42.3) | 0.793 |
| BD Type II | 10 (32.3) | 13 (43.3) | 11 (42.3) |  | 9 (28.1) | 17 (51.5) | 8 (36.4) |  | 10 (37.0) | 9 (26.5) | 15 (57.7) |  |
| Predominant polarity | | | | | | | | | | | | |
| None | 5 (16.1) | 6 (20.0) | 7 (26.9) | 0.853 | 7 (21.9) | 6 (18.2) | 5 (22.7) | 0.746 | 6 (22.2) | 5 (14.7) | 7 (26.9) | 0.376 |
| Depressive | 24 (77.4) | 21 (70.0) | 17 (65.4) |  | 24 (75.0) | 23 (69.7) | 15 (68.2) |  | 20 (74.1) | 24 (70.6) | 18 (69.2) |  |
| Manic | 2 (6.5) | 3 (10.0) | 2 (7.7) |  | 1 (3.1) | 4 (12.1) | 2 (9.1) |  | 1 (3.7) | 5 (14.7) | 1 (3.8) |  |
| Polarity upon entrance to the study | | | | | | | | | | | | |
| Euthymic | 18 (58.1) | 17 (56.7) | 21 (80.8) | 0.177 | 19 (59.4) | 19 (57.6) | 18 (81.8) | 0.179 | 15 (55.6) | 21 (61.8) | 20 (76.9) | 0.157 |
| Depressive | 12 (38.7) | 13 (43.3) | 4 (15.4) |  | 12 (37.5) | 14 (42.4) | 3 (13.6) |  | 10 (37.0) | 13 (38.2) | 6 (23.1) |  |
| Manic/hypomanic | 1 (3.2) | 0 (0.0) | 1 (3.8) |  | 1 (3.1) | 0 (0.0) | 1 (4.5) |  | 2 (7.4) | 0 (0.0) | 0 (0.0) |  |
| Rapid cycling | 6 (19.4) | 5 (16.7) | 3 (11.5) | 0.722 | 7 (21.9) | 3 (9.1) | 4 (18.2) | 0.357 | 5 (18.5) | 5 (14.7) | 4 (15.4) | 0.916 |
| History of psychotic symptoms | 14 (45.2) | 14 (46.7) | 10 (38.5) | 0.809 | 14 (43.8) | 12 (36.4) | 12 (54.5) | 0.412 | 11 (40.7) | 18 (52.9) | 9 (34.6) | 0.342 |
| History of suicide attempts | 11 (35.5) | 6 (20.0) | 4 (15.4) | 0.170 | 10 (31.2) | 7 (21.2) | 4 (18.2) | 0.481 | 11 (40.7) | 4 (11.8) | 6 (23.1) | **0.015** |
| History of admissions | 21 (67.7) | 10 (33.3) | 14 (53.8) | **0.026** | 19 (59.4) | 16 (48.5) | 10 (45.5) | 0.539 | 17 (63.0) | 16 (47.1) | 12 (46.2) | 0.371 |
| Co-morbid psychiatric diagnosis(es) | 29 (93.5) | 19 (63.3) | 19 (73.1) | **0.006** | 28 (87.5) | 21 (63.6) | 18 (81.8) | 0.061 | 21 (77.8) | 29 (85.3) | 17 (65.4) | 0.191 |
| Co-morbid physical diagnosis(es) | 10 (32.3) | 12 (40.0) | 7 (26.9) | 0.578 | 12 (37.5) | 11 (33.3) | 6 (27.3) | 0.736 | 11 (40.7) | 10 (29.4) | 8 (30.8) | 0.613 |
| Family history | 31 (100.0) | 29 (96.7) | 24 (92.3) | 0.284 | 32 (100.0) | 31 (93.9) | 21 (95.5) | 0.387 | 27 (100.0) | 32 (94.1) | 25 (96.2) | 0.453 |
| Clinical course of illness | | | | | | | | | | | | |
| Chronic | 6 (19.4) | 1 (3.3) | 6 (23.1) | 0.164 | 6 (18.8) | 3 (9.1) | 4 (18.2) | 0.681 | 7 (25.9) | 2 (5.9) | 4 (15.4) | 0.265 |
| Episodic | 11 (35.5) | 17 (56.7) | 12 (46.2) |  | 15 (46.9) | 17 (51.5) | 8 (36.4) |  | 11 (40.7) | 16 (47.1) | 13 (50.0) |  |
| Unknown | 14 (45.2) | 12 (40.0) | 8 (30.8) |  | 11 (34.4) | 13 (39.4) | 10 (45.5) |  | 9 (33.3) | 16 (47.1) | 9 (34.6) |  |
| Postpartum depression | 2 (6.5) | 2 (6.7) | 1 (3.8) | 0.883 | 2 (6.2) | 2 (6.1) | 1 (4.5) | 0.961 | 1 (3.7) | 2 (5.9) | 2 (7.7) | 0.822 |
| Antidepressant-induced (hypo)mania | 6 (19.4) | 5 (16.7) | 5 (19.2) | 0.956 | 6 (18.8) | 7 (21.2) | 3 (13.6) | 0.775 | 6 (22.2) | 6 (17.6) | 4 (15.4) | 0.805 |
| MADRS upon admission | | | | | | | | | | | | |
| 10+ | 12 (38.7) | 13 (43.3) | 4 (15.4) | 0.063 | 12 (37.5) | 14 (42.4) | 3 (13.6) | 0.070 | 10 (37.0) | 13 (38.2) | 6 (23.1) | 0.414 |
| 10- | 19 (61.3) | 17 (56.7) | 22 (84.6) |  | 20 (62.5) | 19 (57.6) | 19 (86.4) |  | 17 (63.0) | 21 (61.8) | 20 (76.9) |  |
| YRMS upon admission | | | | | | | | | | | | |
| 10+ | 1 (3.2) | 0 | 1 (3.8) | 0.576 | 1 (3.1) | 0 (0.0) | 1 (4.5) | 0.505 | 2 (7.4) | 0 (0.0) | 0 (0.0) | 0.103 |
| 10- | 30 (96.8) | 30 (100.0) | 25 (96.2) |  | 31 (96.9) | 33 (100.0) | 21 (95.5) |  | 25 (92.6) | 34 (100.0) | 26 (100.0) |  |
| Pharmacotherapy | | | | | | | | | | | | |
| Mood stabilizer monotherapy | 2 (6.5) | 0 | 3 (11.5) | 0.148 | 2 (6.2) | 1 (3.0) | 2 (9.1) | 0.697 | 1 (3.7) | 1 (2.9) | 3 (11.5) | 0.430 |
| Antipsychotic monotherapy | 1 (3.2) | 0 | 0 |  | 1 (3.1) | 0 | 0 |  | 1 (3.7) | 0 | 0 |  |
| Antidepressant monotherapy | 0 | 1 (3.3) | 0 |  | 0 | 1 (3.0) | 0 |  | 0 (0.0) | 0 | 1 (3.8) |  |
| Combination | 28 (90.3) | 29 (96.7) | 21 (80.8) |  | 29 (90.6) | 30 (90.9) | 19 (86.4) |  | 25 (92.6) | 32 (94.1) | 21 (80.8) |  |
| None | 0 | 0 | 2 (7.7) |  | 0 | 1 (3.0) | 1 (4.5) |  | 0 | 1 (2.9) | 1 (3.8) |  |

BD: Bipolar disorder; MADRS: Montgomery-Asberg Depression Rating Scale; YMRS: Young Mania Rating Scale
